# Flow karyotyping of wheat-*Aegilops* additions facilitate dissecting the genomes of *Ae. biuncialis* and *Ae. geniculata* into individual chromosomes

**DOI:** 10.3389/fpls.2022.1017958

**Published:** 2022-10-03

**Authors:** Mahmoud Said, Petr Cápal, András Farkas, Eszter Gaál, László Ivanizs, Bernd Friebe, Jaroslav Doležel, István Molnár

**Affiliations:** ^1^ Institute of Experimental Botany of the Czech Academy of Sciences, Centre of the Region Haná for Biotechnological and Agricultural Research, Olomouc, Czechia; ^2^ Field Crops Research Institute, Agricultural Research Centre, Cairo, Egypt; ^3^ Agricultural Institute, Centre for Agricultural Research, Eötvös Lóránd Kutatási Hálózat (ELKH), Martonvásár, Hungary; ^4^ Wheat Genetics Resource Center, Kansas State University, Manhattan, KS, United States

**Keywords:** *Aegilops biuncialis*, *Aegilops geniculata*, flow karyotyping, chromosome flow sorting, genome dissecting

## Abstract

Breeding of wheat adapted to new climatic conditions and resistant to diseases and pests is hindered by a limited gene pool due to domestication and thousands of years of human selection. Annual goatgrasses (*Aegilops* spp.) with M and U genomes are potential sources of the missing genes and alleles. Development of alien introgression lines of wheat may be facilitated by the knowledge of DNA sequences of *Aegilops* chromosomes. As the *Aegilops* genomes are complex, sequencing relevant *Aegilops* chromosomes purified by flow cytometric sorting offers an attractive route forward. The present study extends the potential of chromosome genomics to allotetraploid *Ae. biuncialis* and *Ae. geniculata* by dissecting their M and U genomes into individual chromosomes. Hybridization of FITC-conjugated GAA oligonucleotide probe to chromosomes suspensions of the two species allowed the application of bivariate flow karyotyping and sorting some individual chromosomes. Bivariate flow karyotype FITC vs. DAPI of *Ae. biuncialis* consisted of nine chromosome-populations, but their chromosome content determined by microscopic analysis of flow sorted chromosomes indicated that only 7M^b^ and 1U^b^ could be sorted at high purity. In the case of *Ae. geniculata*, fourteen chromosome-populations were discriminated, allowing the separation of nine individual chromosomes (1M^g^, 3M^g^, 5M^g^, 6M^g^, 7M^g^, 1U^g^, 3U^g^, 6U^g^, and 7U^g^) out of the 14. To sort the remaining chromosomes, a partial set of wheat*-Ae. biuncialis* and a whole set of wheat-*Ae. geniculata* chromosome addition lines were also flow karyotyped, revealing clear separation of the GAA-rich *Aegilops* chromosomes from the GAA-poor A- and D-genome chromosomes of wheat. All of the alien chromosomes represented by individual addition lines could be isolated at purities ranging from 74.5% to 96.6% and from 87.8% to 97.7%, respectively. Differences in flow karyotypes between *Ae. biuncialis* and *Ae. geniculata* were analyzed and discussed. Chromosome-specific genomic resources will facilitate gene cloning and the development of molecular tools to support alien introgression breeding of wheat.

## Introduction

The gene pool of bread wheat has narrowed during thousands of years of domestication and cultivation. This resulted in the loss of some useful genes, including those underlying resistance to diseases, pests, and abiotic stress as well as those affecting grain quality. Reduced genetic diversity hampers the development of new wheat cultivars with improved quality and stress tolerance. As the wild-crop relatives have not been subjected to human selection, they exhibit large genetic variation and offer a rich source of alleles and genes for crop improvement (Tanksley and McCouch, 1997; Feuillet et al., 2008). Alien gene transfer by chromosome engineering is an effective strategy to exploit this wild genetic diversity for wheat improvement (Schneider and Molnár-Láng, 2009; Ochoa et al., 2015; Zhang et al., 2017).

The tribe Triticeae contains about one hundred annual species, including those of the genus *Aegilops*, commonly referred to as goatgrasses (van Slageren, 1994). Annual goatgrasses (*Aegilops* spp.) are closely related to the genus *Triticum* and represent a rich source of genes with considerable agronomic value (Friebe et al., 1996; Schneider et al., 2008; Kilian et al., 2011). Within the genus, seven different genomes (C, D, M, N, S, T, and U) were identified in eleven diploid and twelve polyploid species (van Slageren, 1994). Among the allopolyploid *Aegilops* species containing M- and U-genomes are *Ae. biuncialis* Vis. (2n = 4x = 28, U^b^U^b^M^b^M^b^) and *Ae. geniculata*. Roth. (2n = 4x = 28, U^g^U^g^M^g^M^g^) exhibits a wide eco-geographical distribution in the Mediterranean and Western Asiatic regions (van Slageren, 1994). The wide distribution of these species has been attributed to their intraspecific diversity manifested at DNA sequence level (Arrigo et al., 2010; Ivanizs et al., 2019; Ivanizs et al., 2022), as well as chromosome level structural changes in the genebank collections of *Ae. biuncialis* and *Ae. geniculata* (Badaeva et al., 2004; Molnár et al., 2011a). In parallel with the genetic diversity, these allotetraploid species have been reported as sources of resistance to rust diseases (Olivera et al., 2018), powdery mildew, Hessian fly, and greenbug (Gill et al., 1985). Other accessions have high tolerance to abiotic stresses such as frost (Ekmekci and Terzioglu, 2002), salt (Darko et al., 2020), and drought (Molnár et al., 2004; Pradhan et al., 2012; Dulai et al., 2014), or found to have beneficial grain quality traits, such as high micronutrient (Farkas et al., 2014), edible fiber and protein content compared to bread wheat (Rakszegi et al., 2017; Marcotuli et al., 2019; Ivanizs et al., 2022). In order to transfer desirable agronomic traits from these *Aegilops* species, a partial set of wheat-*Ae. biuncialis* chromosome addition lines (2M^b^, 3M^b^, 7M^b^, 1U^b^, 2U^b^, and 3U^b^) (Schneider et al., 2005), and a complete set of wheat-*Ae. geniculata* addition lines were developed (Friebe et al., 1999). In contrast to the potential allelic variations for useful traits, the genetic diversity of *Ae. biuncialis* and *Ae. geniculata* remains largely unexploited in wheat breeding, mainly because the selection of alien introgression lines is laborious and time-consuming (Kuraparthy et al., 2009; Farkas et al., 2014; Tiwari et al., 2014).

One way to facilitate the development of wheat-*Aegilops* translocation lines is to sequence M and U genomes. Assembled chromosome sequences and gene models would provide unique information for the development of DNA markers suitable for selection of wheat lines with introgressed *Aegilops* fragments (Tiwari et al., 2014; Tiwari et al., 2015; Li et al., 2021; Said et al., 2021); for mining wild alleles of agronomically important genes (Ivanizs et al., 2022); and for cloning resistance genes against pests and diseases (Yu et al., 2022). However, sequencing and assembly of large and allotetraploid genomes of *Aegilops* remain challenging because of the high proportion of repetitive DNA and allopolyploid genome structure. In fact, the 1C genome sizes of *Ae. biuncialis* and *Ae. geniculata* were estimated to be 10,142 Mbp (10.37 pg DNA) and 10,064 Mbp (10.29 pg DNA), respectively (Eilam et al., 2008; Bennett and Leitch, 2011).

The analysis of large and complex nuclear genomes can be simplified by dissecting them into individual chromosomes by flow cytometric sorting (Doležel et al., 2007; Doležel et al., 2014). Chromosome fractions purified by flow sorting can be used as DNA templates for PCR-based assays, including the assignment of wheat molecular markers to chromosomes of *Aegilops* and the establishment of wheat-alien homoeologous relationships (Molnár et al., 2011a; Molnár et al., 2016). Chromosomal DNA has also been reported to be suitable for the production of chromosome specific BAC libraries and for the development of molecular and cytogenetic markers (Šimková et al., 2008a; Doležel et al., 2012; Doležel et al., 2014; Kapustová et al., 2019). Barley, rye, and wheat flow sorted chromosomes facilitated the establishment of linear gene-order models and allowed assessment of gene synteny with other species (Mayer et al., 2009; Mayer et al., 2011; Martis et al., 2013; International Wheat Genome Sequencing Consortium (IWGSC), 2014; Molnár et al., 2016). Moreover, the development of reference genome assemblies for agronomically important species such as barley (International Barley Genome Sequencing Consortium et al., 2012) and bread wheat (International Wheat Genome Sequencing Consortium (IWGSC), 2018), and for their wild gene source species, *Ae. sharonensis* (Yu et al., 2022) was enabled through the use of flow sorted chromosomes. Chromosome-based approaches were also developed for cloning agronomically important genes (Sánchez-Martín et al., 2016; Thind et al., 2017; Zwyrtková et al., 2021) and have become an important tool for the functional genomics of cereals.

The application of chromosome-based genomic approaches relies on the ability to discriminate individual chromosomes by flow cytometric analysis. Original protocols developed for *Vicia faba* used univariate flow karyotyping, where intact mitotic chromosomes are classified according to the fluorescence intensity of a fluorochrome bound to DNA, most frequently DAPI, which provides information about chromosome size or relative DNA content (Doležel et al., 2014). In the case of cereals, similar to most plant species, the size of individual chromosomes is quite similar, and only one to three chromosomes show enough difference to form specific peaks on flow karyotypes, while the remaining chromosomes form either a single composite peak, or several smaller composite peaks. In the case of *Aegilops* with M and U genomes, univariate flow karyotyping indicated that three chromosomes (1U, 3U, and 6U) could be sorted in high purity from diploid *Ae. umbellulata* (2n = 4x = 14, UU), two (7M^b^ and 1U^b^) from *Ae. biuncialis*, while no chromosome specific peaks were detected in *Ae. comosa* (2n = 4x = 14, MM) and *Ae. geniculata* (Molnár et al., 2011a).

To overcome the problem of chromosome discrimination, Giorgi et al. (2013) developed a method termed FISHIS (fluorescence *in situ* hybridization in suspension), which uses FITC conjugated microsatellite probes to visualize GAA clusters on DAPI stained chromosomes prior to flow cytometric analysis. The resulting bivariate DAPI vs. FITC flow karyotypes allow much better discrimination of individual chromosomes and sorting of the majority of them at high purity (Giorgi et al., 2013; Doležel et al., 2014; Akpinar et al., 2015; Molnár et al., 2016; Said et al., 2019a; Said et al., 2021). Bivariate flow karyotyping has been used successfully in plant species whose genomes contain GAA clusters in cytogenetically detectable amounts, as demonstrated in *T. aestivum*, *T. turgidum* ssp. *durum*, and *Hordeum vulgare* (Doležel et al., 2021). The M and U genomes of *Aegilops* are also rich in GAA clusters (Molnár et al., 2011a), and bivariate flow cytometric analysis of diploid *Ae. comosa* and *Ae. umbellulata* allowed flow sorting of individual chromosomes 1M–7M and 1U–7U, respectively (Molnár et al., 2016; Said et al., 2021). Chromosomes flow sorted from these two species allowed for the study of wheat-*Aegilops* cross-genome homoeology and the development of molecular tools to support wheat alien chromosome introgression breeding programs (Said et al., 2021). On the other hand, bivariate flow karyotyping has not been applied to allotetraploid *Aegilops* species so far.

Another approach to discriminating and flow sorting individual chromosomes is to use wheat-alien cytogenetic stocks, such as translocations, disomic chromosome additions, substitutions, or ditelosomic lines (Doležel et al., 2007; Doležel et al., 2014). If the size of the chromosomes containing the alien chromatin differs from that of normal wheat chromosomes, it can be discriminated against and flow sorted at high purity. The availability of wheat-alien addition lines made it possible to flow sort chromosomes of *Dasypyrum villosum* (Grosso et al., 2012; Xing et al., 2021). This approach was more effective if FISHIS was applied, as reported for sorting chromosome arms of 2US, 2UL, and 7UL from wheat-*Ae. umbellulata* ditelosomic lines (Molnár et al., 2016). The application of wheat-alien chromosome addition lines was especially important when no individual chromosomes could be resolved in the bivariate flow karyotype of the gene source species, as was the case of *Agropyron cristatum*, whose chromosomes 1P–6P could only be flow sorted at high purity from wheat-*A. cristatum* chromosome addition lines (Said et al., 2019a).

Motivated by the need to develop chromosome-specific genomic resources for structural and functional analysis of *Ae. biuncialis* and *Ae. geniculata* and to produce molecular tools to facilitate gene introgression into wheat, we investigated the ability to flow sort individual chromosomes 1M–7M and 1U–7U of the *Ae. biuncialis* and *Ae. geniculata* genomes. We used FITC-conjugated (GAA)_7_ microsatellite repeat probe for fluorescent labeling of DAPI stained mitotic chromosomes in suspension prior to flow-cytometric analysis to potentially facilitate sorting individual M and U chromosomes from tetraploid *Ae. biuncialis* and *Ae. geniculata* species as well as from wheat-*Aegilops* addition lines.

## Materials and methods

### Plant material

Seeds of *Ae. geniculata* (2*n* = 4*x* = 28, U^g^U^g^M^g^M^g^) accessions AE1311/00 and AE660/83 were obtained from the Leibniz Institute of Plant Genetics and Crop Plant Research, IPK (Gatersleben, Germany). *Ae. biuncialis* (2*n* = 4*x* = 28, U^b^U^b^M^b^M^b^) accession MvGB382, bread wheat line Mv9kr1 and Mv9kr1-*Ae. biuncialis* accession MvGB642 chromosome addition lines 2M^b^, 3M^b^, 7M^b^, 1U^b^, and 6U^b^ (double addition) and 3U^b^ (Schneider et al., 2005) were obtained from the Martonvásár Cereal Genebank (Martonvásár, Hungary). Bread wheat cv. Chinese Spring (CS) and CS-*Ae. geniculata* accession TA2899 chromosome addition lines 1M^g^–7M^g^ and 1U^g^–7U^g^ (Friebe et al., 1999) were kindly provided by Dr. Bernd Friebe (Kansas State University, Manhattan, Kansas, USA). The information about the plant material used in this work is summarized in **Supplementary Table 1**.

### Mitotic chromosome preparations

For the flow cytometric analysis and chromosome sorting, suspensions of intact mitotic metaphase chromosomes were prepared from the synchronized root tips of allotetraploid *Ae. biuncialis* and *Ae. geniculata*, and hexaploid wheat genotypes Mv9kr1, CS, and Mv9kr1-*Ae. biuncialis* and CS-*Ae. geniculata* chromosome addition lines following the method of Vrána et al. (2016a, 2016b). Briefly, root tip meristem cells of young seedlings were synchronized using hydroxyurea, accumulated in metaphase using amiprophos-methyl and mildly fixed by formaldehyde as described by Vrána et al. (2016a, 2016b). Mitotic metaphase chromosomes were released from root tips into LB01 buffer by mechanical homogenization.

### Flow cytometric analysis and sorting of *Aegilops* chromosomes

Prior to flow cytometric analysis, chromosomes in suspension were fluorescently labeled by FISHIS using oligonucleotide probe 5’-FITC-(GAA)_7_-FITC-3’ (Integrated DNA Technologies, Inc., Iowa, USA) and counterstained with DAPI (4´,6-diamidino 2-phenylindole) following Giorgi et al. (2013). Bivariate flow karyotyping and chromosome sorting were done on the FACSAria II SORP flow cytometer and sorter (Becton Dickinson Immunocytometry Systems, San José, USA) as described by Molnár et al. (2016) and Said et al. (2019a). Chromosome samples were analyzed at rates of 900–1,400 particles per second. Bivariate flow karyotypes of FITC pulse area (FITC-A) vs. DAPI pulse area (DAPI-A) fluorescence were acquired and 20,000 events were recorded to create a bivariate flow karyotype for each experiment. Sort regions were set on the flow karyotypes and chromosomes were flow sorted at rates of 12–24 per second. Chromosome content in flow-sorted fractions and identification of contaminating chromosomes were determined by fluorescence *in situ* hybridization (FISH) on chromosomes sorted onto microscope slides using probes for *pSc*119.2, *Afa* family repeat, and 45S rDNA according to Molnár et al. (2016). Briefly, 3,000 chromosomes were flow sorted from each chromosome population onto a microscope slide into a 3 μl drop of PRINS buffer supplemented with 2.5% sucrose (Kubaláková et al., 1997). The slides were air dried and used for FISH experiments. The chromosomes were classified following the karyotype described by Molnár et al. (2011a).

### Preparation of probes for FISH

The rye 120-bp repeat family (Bedbrook et al., 1980) was amplified by PCR from DNA clone *pSc*119.2 using M13 universal primers (Said et al., 2018), whereas *Afa* family repeat was amplified from genomic DNA of bread wheat CS using primers AS-A and AS-B (Nagaki et al., 1995). The probes *pSc*119.2 and *Afa* family repeat were labeled by PCR with digoxigenin-dUTP and biotin-dUTP (Roche, Mannheim, Germany), respectively (Said et al., 2018; Said et al., 2019b). Plasmid pTa71 (45S rDNA) containing a 9-kb fragment from *T. aestivum* with 18S-5.8S-26S rDNA and intergenic spacers (Gerlach and Bedbrook, 1979) was labeled by nick translation with either biotin-dUTP or digoxigenin-dUTP (Roche) using standard Nick Translation Mix kits (Roche) following the instructions of the manufacturer.

### Fluorescence *in situ* hybridization

All FISH analyses of chromosomes sorted onto microscope slides were done with three probes (45S rDNA, tandem repeats *pSc*119.2 and *Afa* family), which were hybridized simultaneously. The experiments, including the denaturation of probe and chromosomal DNA, the hybridization, and the post hybridization washes, followed the protocols of Molnár et al. (2016) and Said et al. (2021) with small modifications. Briefly, the FISH hybridization mix (15 μl per slide) contained 50 ng labeled probe DNA, 50% v/v formamide, 2 × SSC (0.15 mol/l NaCl plus 0.015 mol/l sodium citrate), 10% w/v dextran sulfate, 0.4 μg salmon sperm DNA, and 0.1% w/v sodium dodecyl sulfate. The probes were denatured at 93°C for 10 min and maintained in ice for 5 min. Then the chromosomes and probes were denatured together at 80°C for 45 s under high moisture conditions. Hybridization was carried out overnight at 37°C in a humid chamber. Hybridization signals of digoxigenin-labeled probes were detected using anti-digoxigenin fluorescein isothiocyanate (Roche), while the signals of biotin-labeled probes were detected with Cy3-conjugated streptavidin (Invitrogen, Life Technologies, Carlsbad, USA). Chromosomes were mounted and counterstained with DAPI in an antifade mounting medium (Vectashield, Vector Laboratories, Burlingame, USA).

### Microscopy, software, signal capture and image analysis

After FISH, chromosomes on microscope slides were examined using an Axio Imager Z.2 fluorescence microscope (Zeiss, Oberkochen, Germany) equipped with a Cool Cube 1 camera (Metasystems, Altlussheim, Germany) and appropriate filter sets. The signal capture and picture processing were performed using ISIS software (Metasystems). The final image adjustment was done in Adobe Photoshop CS5 (Adobe Systems Incorporated, San Jose, USA). In order to determine the chromosome content of the flow sorted samples, at least 100 chromosomes were identified for each sample using a standard karyotype (Molnár et al., 2011a).

## Results

### Chromosome flow sorting from tetraploid *Ae. biuncialis* and *Ae. geniculata*

We first investigated the potential of univariate flow cytometric analysis based on DAPI fluorescence alone to flow sort individual chromosomes from allotetraploid *Ae. biuncialis* and *Ae. geniculata*. The analyses showed that peaks of some chromosomes (7M^b^, 1U^b^), (5M^g^, 6M^g^, 1U^g^), and (4M^g^, 5M^g^, 6M^g^, 1U^g^) from *Ae. biuncialis* MvGB382, *Ae. geniculata* 1,311/00, and *Ae. geniculata* AE660/83, respectively, could be discriminated and the chromosomes flow sorted individually (**Supplementary Figure 1**). The remaining chromosomes could be sorted only into groups of more than two.

**Figure 1:**
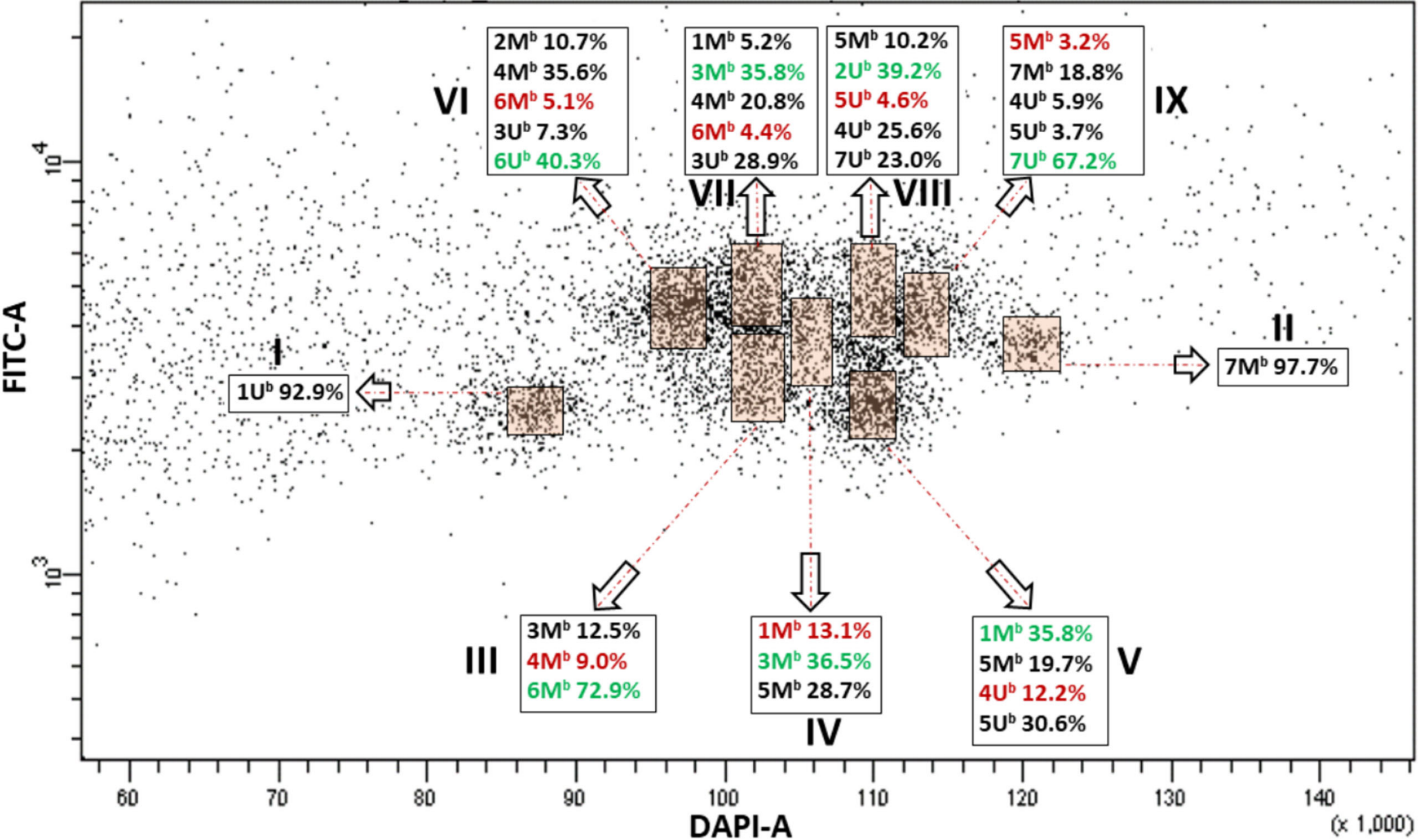
Bivariate flow karyotype (DAPI-A vs. FITC-A) of *Ae. biuncialis* acc. MvGB382. Flow cytometric analysis of DAPI stained mitotic chromosomes labeled by FISHIS with GAA_7_-FITC probe discriminated nine chromosome populations (I–IX) representing the whole genome of the species. Chromosomes in the sort windows (brown) were sorted onto microscope slides and classified by FISH. The red and green numbers within the chromosome content of the sorted populations (black rectangles) refer to the lowest and the highest purities, respectively. x-axis, relative DAPI fluorescence. y-axis; relative GAA_7_-FITC fluorescence.

We then applied bivariate flow cytometric analysis to improve the resolution of individual chromosomes of *Ae. biuncialis* and *Ae. geniculata*. FISHIS labeling of (GAA)_7_ clusters on the DAPI stained chromosomes resulted in a bivariate (DAPI-A vs. FITC-A) flow karyotype of *Ae. biuncialis* acc. MvGB382. On the dot plot flow karyotype (**Figure 1**), nine chromosome groups (I–IX) were discriminated, which is less than the haploid chromosome number (n = 14) of the species.

**Figure 2:**
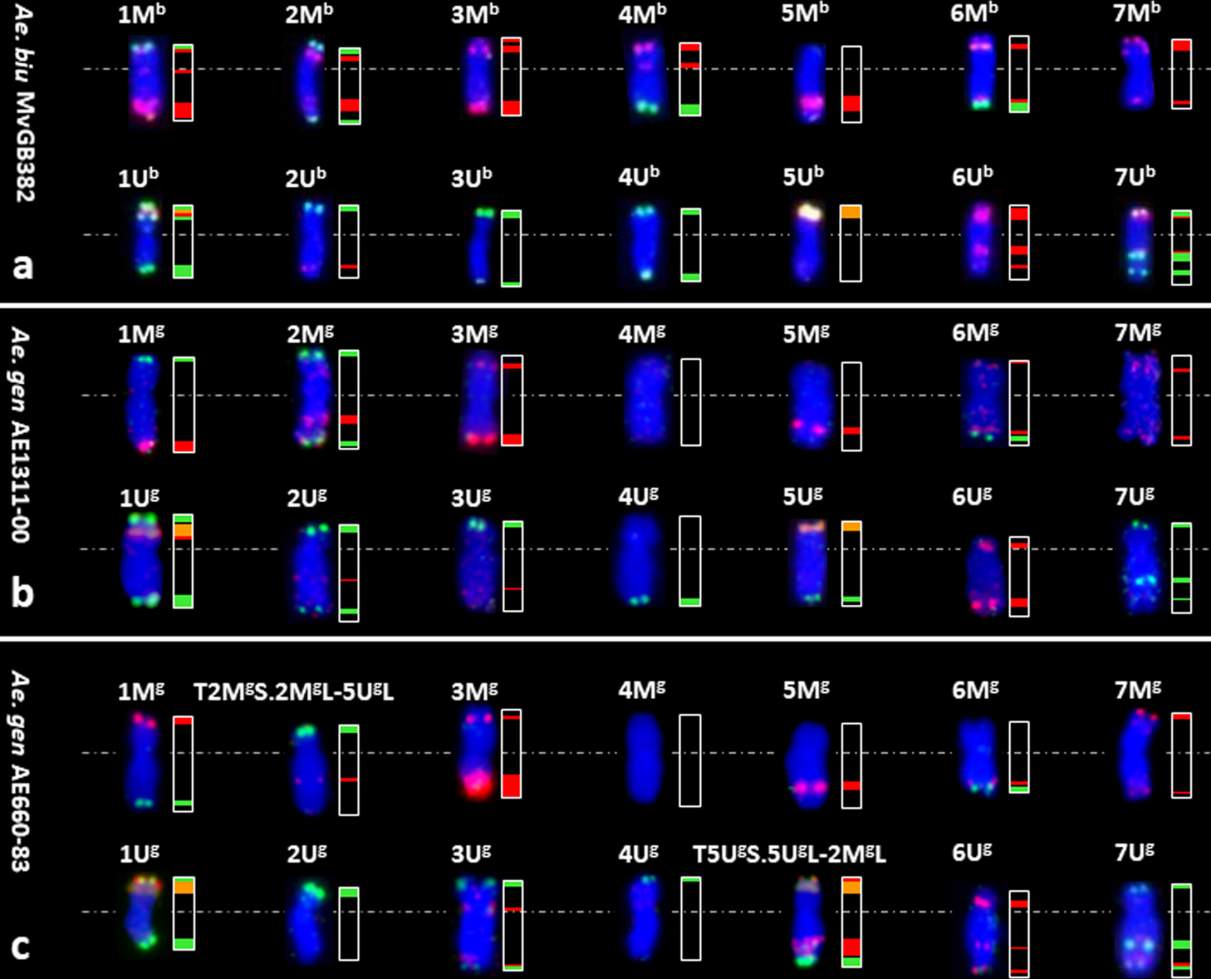
Identification of the M- and U-genome chromosomes (left) flow sorted from *Aegilops* species and their Idiograms (right). FISH with probes for *Afa* family repeat (red), 45S rDNA (orange) and *pSc*119.2 repeat (green) on chromosomes flow sorted from *Ae. biuncialis* acc. MvGB382 **(A)**, *Ae. geniculata* acc. AE1311/00 **(B)** and *Ae. geniculata* acc. AE660/83 **(C)**. Chromosomes were counterstained with DAPI (blue).

FISH using probes for 45S rDNA, tandem repeats *pSc*119.2 and the *Afa* family on chromosomes flow sorted onto microscope slides enabled determination of the chromosome content of the nine groups of the flow karyotype (**Figures 1**, **2A**). The analysis showed that chromosomes 1U^b^ (I) and 7M^b^ (II) could be sorted individually at purities reaching 92.9% and 97.7%, respectively. However, the remaining chromosomes could be flow sorted only into groups of three (III and IV), four (V), or even five (VI–IX) chromosomes. Identification of flow sorted M^b^- and U^b^-genome chromosomes showed that two groups III and IV consisted of each of three chromosomes with proportions equaling 9.0% (4M^b^)–72.9% (6M^b^) and 13.1 (1M^b^)–36.5 (3M^b^), respectively. One group (V) contained four chromosomes with proportions equaling 12.2% (4U^b^)–35.8% (1M^b^). Four groups (VI–IX) were each comprised of five chromosomes with proportions ranged from 5.1% (6M^b^)–40.3% (6U^b^), 4.4% (6M^b^)–35.8% (3M^b^), 4.6% (5U^b^)–39.2% (2U^b^), and 3.2% (5M^b^)–67.2% (7U^b^), respectively (**Figure 1**).

**Figure 3:**
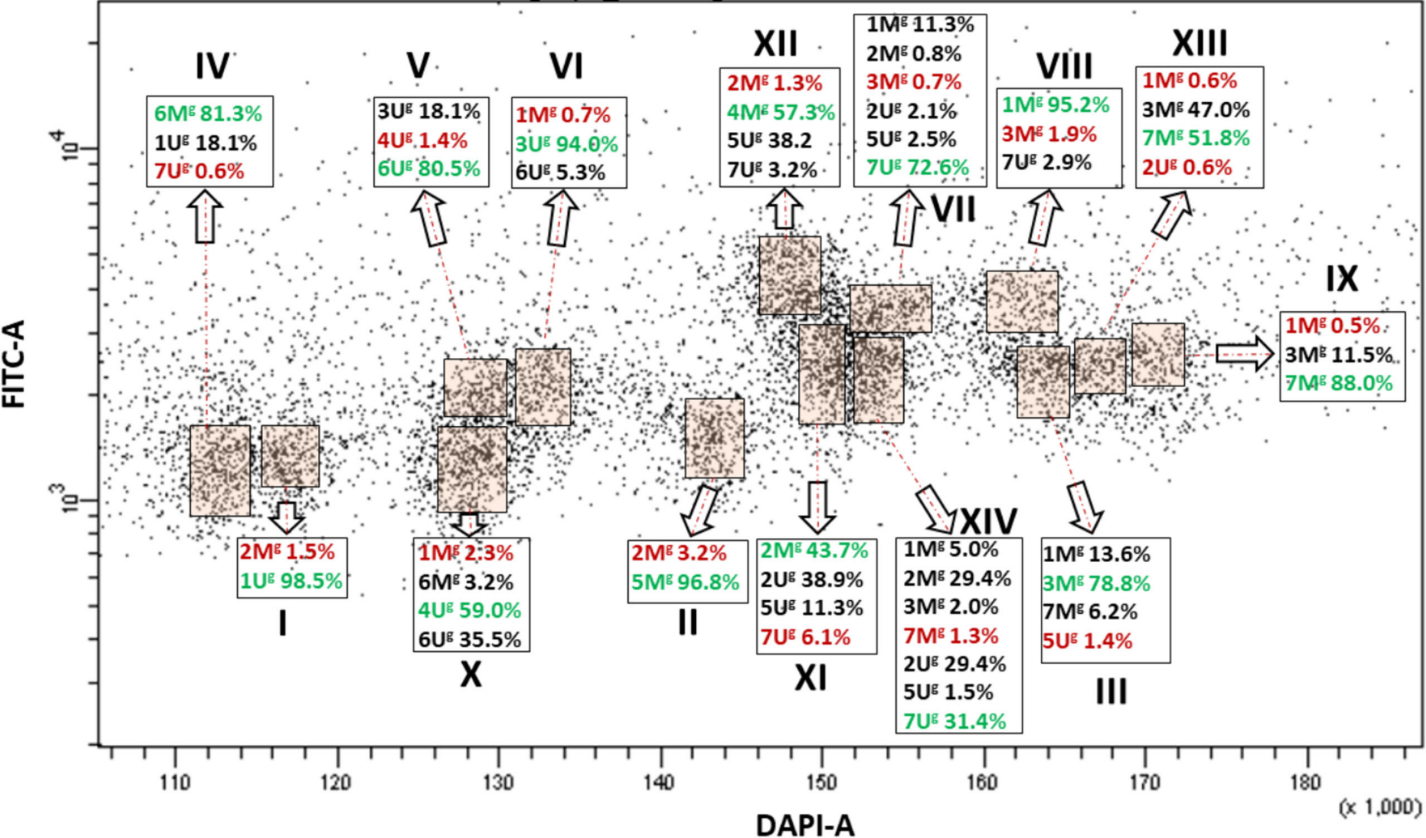
Bivariate flow karyotype (DAPI-A vs. FITC-A) obtained after FISHIS with the GAA_7_ probe on chromosomes isolated from *Ae. geniculata* acc. AE1311/00. The analysis permitted discrimination of fourteen chromosome populations (orange boxes) representing the whole genome of the species. The red and green numbers within a group of chromosomes sorted together refer to the lowest and highest purities, respectively. x-axis, relative DAPI fluorescence; y-axis, relative GAA_7_-FITC fluorescence.

Bivariate (DAPI-A vs. FITC-A) flow karyotype of *Ae. geniculata* accession AE1311/00 allowed identification of fourteen chromosome groups (I–XIV) corresponding to the haploid chromosome number (n = 14) of the species (**Figure 3**). FISH on flow sorted chromosomes (**Figure 2B**) confirmed that nine groups (I–IX) consisted of individual chromosomes (1U^g^, 5M^g^, 3M^g^, 6M^g^, 6U^g^, 3U^g^, 7U^g^, 1M^g^, and 7M^g^), which could be flow sorted separately at purities equaling 98.5%, 96.8%, 78.8%, 81.3%, 80.5%, 94.0%, 72.6%, 95.2%, and 88.0%, respectively. The other four groups (X–XIII) consisted of four chromosomes each and could be flow sorted at proportions ranged from 2.3% (1M^g^)–59.0% (4U^g^), 43.7% (2M^g^)–6.1% (7U^g^), 1.3% (2M^g^)–57.3% (4M^g^), and 0.6% (1M^g^ and 2U^g^)–51.8% (7M^g^), respectively. The remaining group (XIV) was identified as a composite of seven chromosomes, which were flow sorted in proportions ranging from 1.3% (7M^g^) to 31.4% (7U^g^).

**Figure 4:**
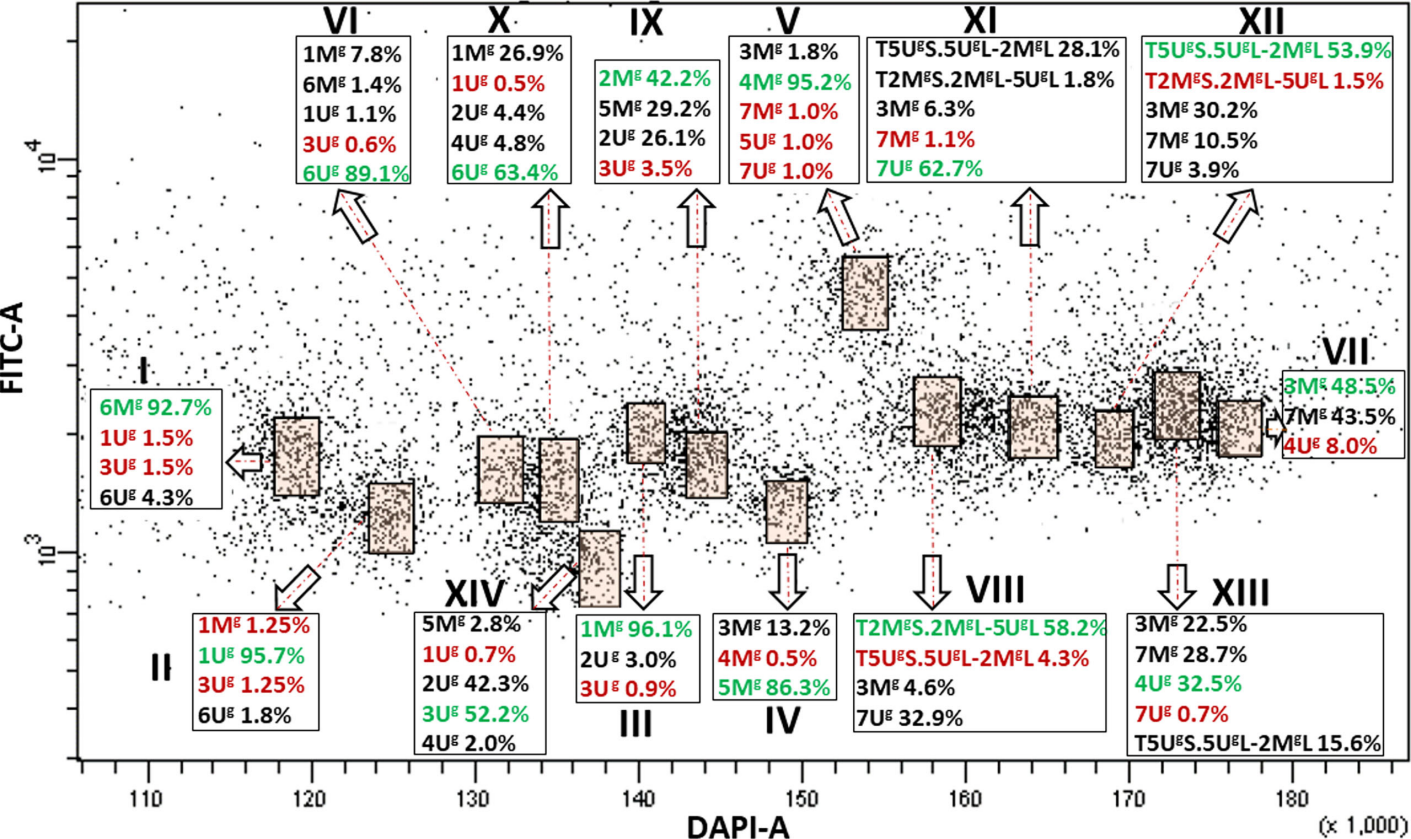
Bivariate flow karyotype (DAPI-A vs. FITC-A) obtained after FISHIS with the GAA_7_ probe on chromosomes isolated from *Ae. geniculata* acc. AE660/83. The analysis permitted discrimination of fourteen chromosome populations (orange boxes) representing the whole genome of the species. The red and green numbers within a group of chromosomes sorted together refer to the lowest and highest purities, respectively. x-axis, relative DAPI fluorescence; y-axis, relative GAA_7_-FITC fluorescence.

We then wanted to check if there was any effect of the modification in the karyotype on flow sorting of individual chromosomes, therefore we investigated the *Ae. geniculata* accession AE660-83, which has a 5U^g^/2M^g^ reciprocal translocation (Molnár et al., 2011a) and as shown in **Figure 2C**. Bivariate (DAPI vs. FITC) flow karyotyping after FISHIS allowed identification of fourteen chromosome groups (I–XIV) representing the whole genome of the species (**Figure 4**). FISH analysis (**Figure 2C**) indicated that six chromosomes (6M^g^, 1U^g^, 1M^g^, 5M^g^, 4M^g^, and 6U^g^) could be sorted individually from groups (I–VI) at reasonable purities of 92.7%, 95.7%, 96.1%, 86.3%, 95.2%, and 89.1%, respectively. The remaining chromosomes could be sorted only into groups of three, four, or five. One group (VII) consisted of three chromosomes, which were sorted in proportions ranging from 8.0% (4U^g^) to 48.5% (3M^g^). Two groups (VIII and IX) contained four chromosomes and could be sorted into proportions ranging from 4.3% (T5U^g^S.5U^g^L-2M^g^L)–58.2% (T2M^g^S.2M^g^L-5U^g^L) and 3.5% (3U^g^)–42.2% (2M^g^), respectively. The remaining groups (X–XIV) consisted of five chromosomes each and flow sorted at proportions ranged 0.5% (1U^g^)–63.4% (6U^g^), 1.1% (7M^g^)–62.7% (7U^g^), 1.5% (T2M^g^S.2M^g^L-5U^g^L)–53.9% (T5U^g^S.5U^g^L-2M^g^L), 32.5% (4U^g^)–0.7% (7U^g^) and 0.7% (1U^g^)–52.2% (3U^g^), respectively.

In both species, the majority of the chromosome groups were contaminated with chromosomes that were assigned to other groups but were also oscillating between the different chromosome populations as shown in **Figures 1**, **3**, **4**.

### Chromosome flow sorting from wheat-*Aegilops* disomic addition lines

**Figure 5:**
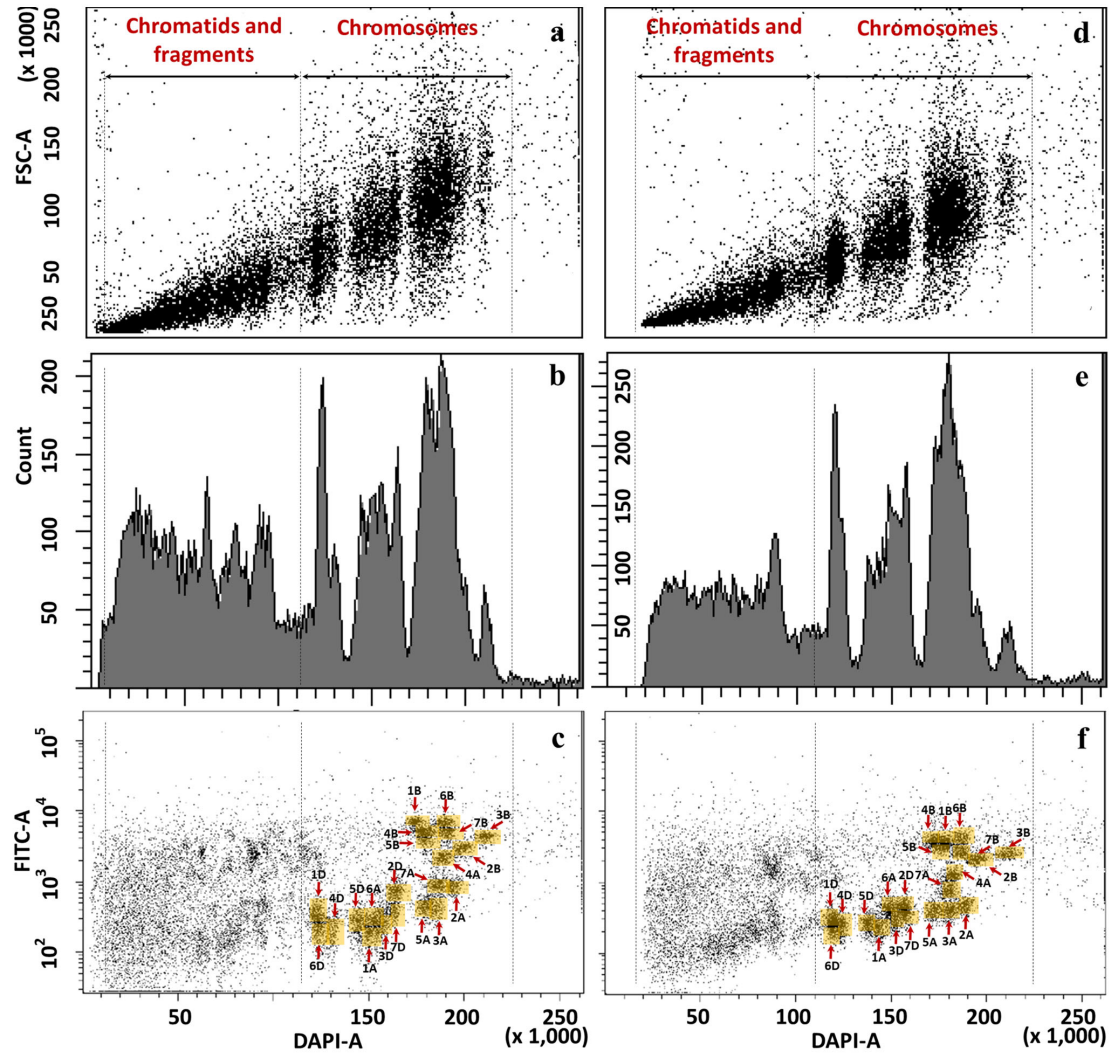
Flow cytometric analysis and sorting in hexaploid wheat cv. Mv9kr1 **(A–C)** and cv. CS **(D–F)** chromosomes. **(A, D)**. bivariate flow karyotypes DAPI (x-axis) vs. FSC (y-axis), **(B, E)** monovariate flow karyotypes DAPI (x-axis) showing count (y-axis), **(C, F)** bivariate flow karyotypes DAPI (x-axis) vs. FITC (y-axis) obtained after FISHIS with (GAA)_7_ on chromosomes isolated from wheat Mv9kr1 **(C)** and wheat CS **(F)**. The analysis permitted discrimination of the 21 chromosome pairs (yellow boxes) of hexaploid wheat.

Because of the isolation of pure (>80%) fractions was not possible for each of the M- and U-genome chromosomes of allotetraploid *Ae. biuncialis* and *Ae. geniculata*, we also tested wheat-*Ae. biuncialis* and wheat-*Ae. geniculata* disomic addition lines. As control, flow cytometric analysis of the parental hexaploid wheat genotypes, Mv9kr1 and CS, was carried out first (**Figure 5**). We tested different settings of the flow cytometer to find the most optimal display of the chromosomal events for discrimination of individual chromosomes. Although the bivariate analysis using the gain of the forward scatter pulse area (FSC-A) and DAPI fluorescence pulse area (DAPI-A) (**Figures 5A, D** **)** and monovariate analysis of DAPI-A and number of chromosomal events (**Figures 5B, E** **)** are essential, they did not enable flow sorting of individual chromosomes.

On the contrary, bivariate flow karyotypes DAPI-A vs. FITC-A obtained from wheat genotypes allowed the resolution of almost all the 21 wheat chromosomes (**Figures 5C, F** **)**. The positions of the 21 chromosome pairs from ABD genomes of both Mv9kr1 and CS genotypes were also assigned (**Figures 5C, F** **)** and confirmed by FISH experiments on chromosomes flow sorted onto microscope slides from each population (data not shown). The position of the chromosomes on the flow karyotype indicated that the D-genome chromosomes have the smallest DNA content, followed by the chromosomes of the A-genome. Because the A- and D-genome chromosomes have a small number of (GAA)_7_ clusters, they were detected at the lower FITC fluorescence intensities. On the contrary, B-genome chromosomes, having the highest DNA content and a huge number of (GAA)_7_ clusters, were detected at the flow karyotype areas characterized by higher DAPI and FITC fluorescence intensities.

**Figure 6:**
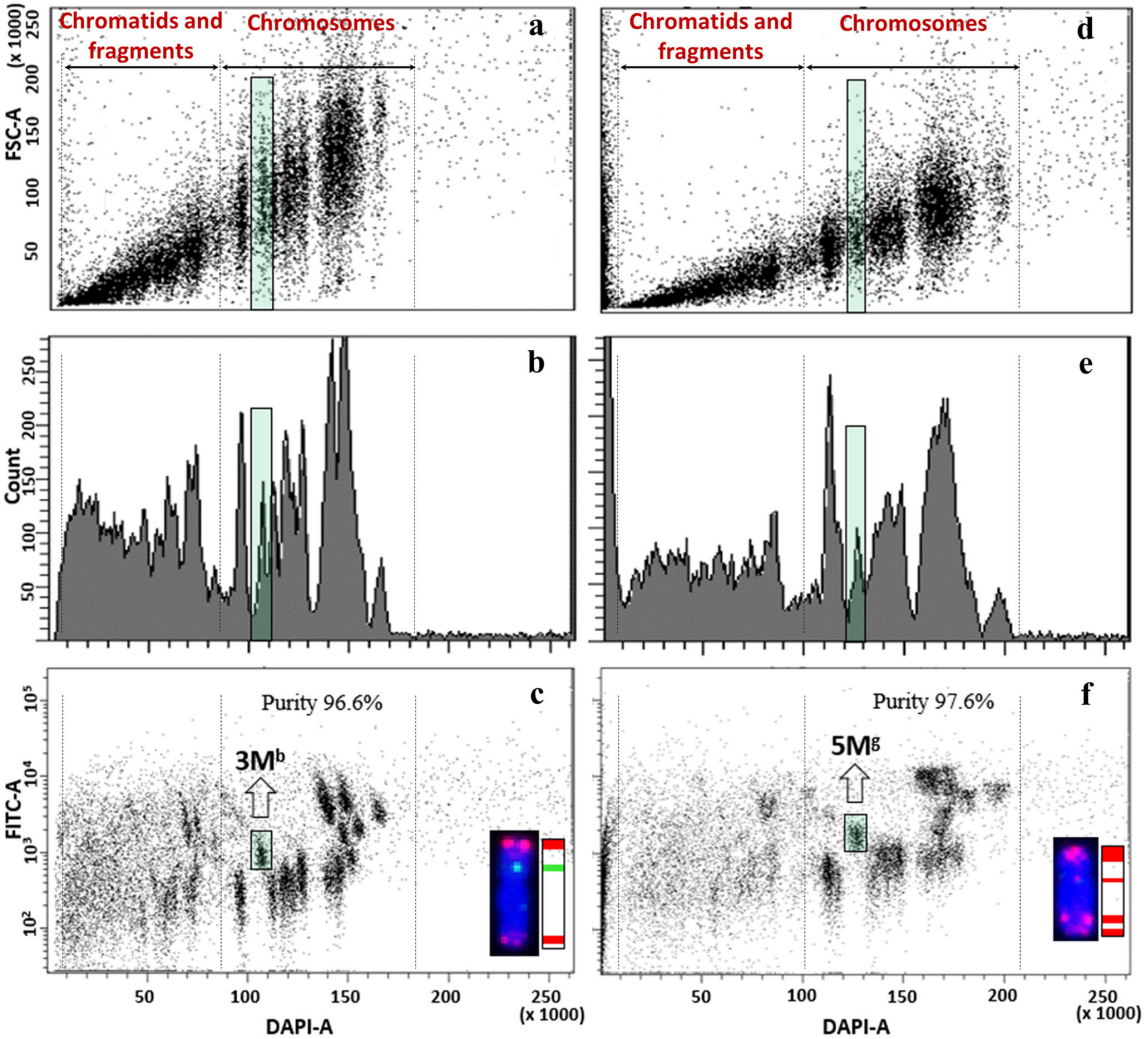
Flow cytometric analysis and sorting of *Ae. biuncialis* **(A-C)** and *Ae. geniculata* **(D–F)** chromosomes from wheat disomic addition lines. **(A, D)**. bivariate flow karyotypes DAPI (x-axis) vs. FSC (y-axis), **(B, E)** monovariate flow karyotypes DAPI (x-axis) showing count (y-axis), **(C, F)** bivariate flow karyotypes DAPI (x-axis) vs. FITC (y-axis) obtained after FISHIS with (GAA)_7_ on chromosomes isolated from wheat Mv9kr1-*Ae. biuncialis* acc. MvGB642 **(C)** and wheat CS-*Ae. geniculata* acc. TA2899 **(F)** addition lines. The analysis permitted discrimination and sorting of the tetraploid *Aegilops* chromosomes (green boxes) in the genetic background of bread wheat **(C, F)**. The chromosomes (inset) were sorted at high purity and identified by FISH on microscope slides with probes for *Afa* family repeat (red), 45S rDNA (orange) and *pSc*119.2 repeat (green). Chromosomes were counterstained with DAPI (blue).

**Figure 7:**
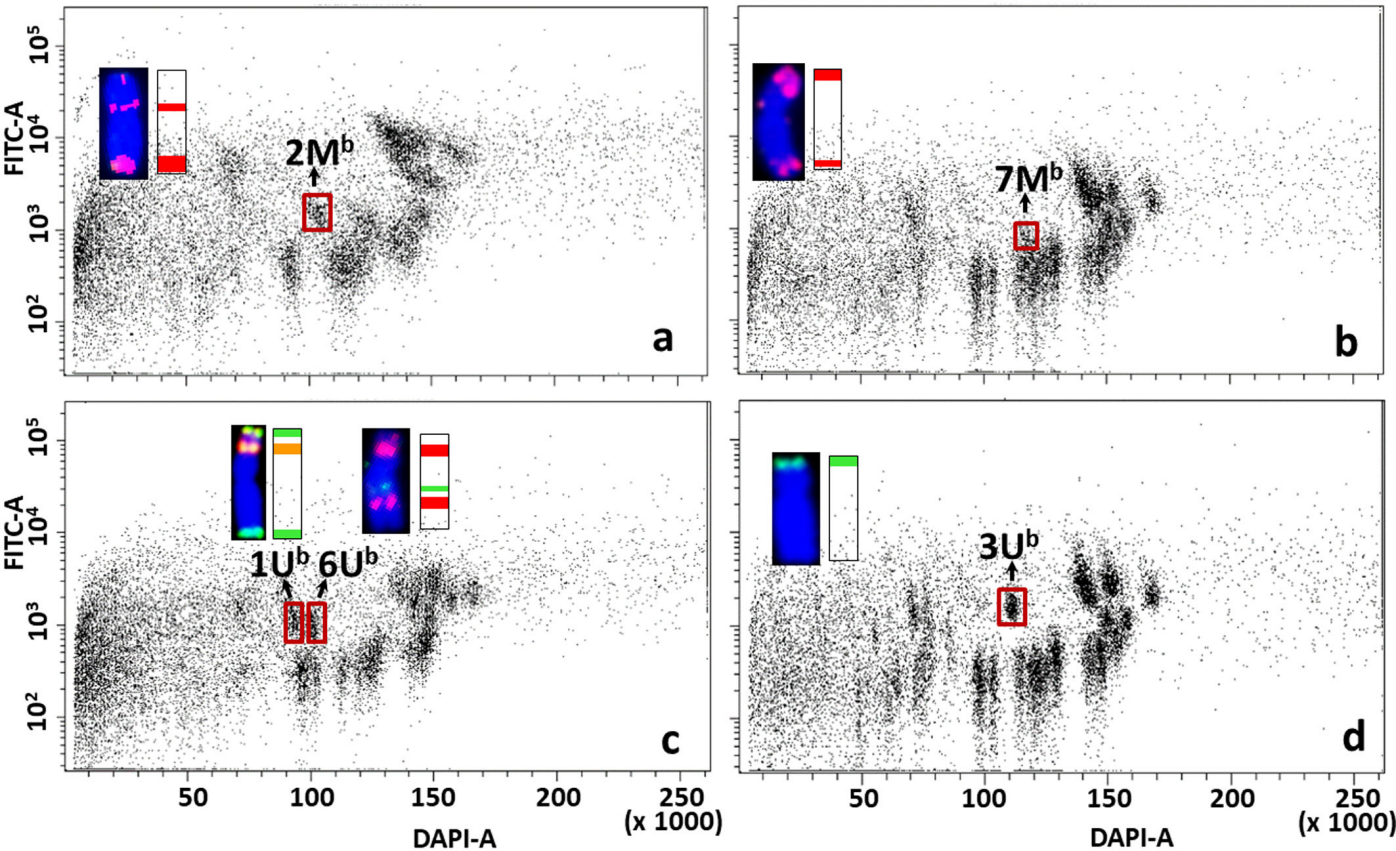
Bivariate flow karyotypes DAPI vs. FITC obtained after FISHIS with (GAA)_7_ on chromosomes isolated from wheat Mv9kr1-*Ae. biuncialis* acc. MvGB642 addition lines. The analysis permitted discrimination of chromosomes from tetraploid *Ae. biuncialis* (brown boxes) in the genetic background of bread wheat **(A–D)**. The chromosomes (inset) were sorted in high purity and identified by FISH on microscope slides with probes for *Afa* family repeat (red), 45S rDNA (orange) and *pSc119*.2 repeat (green). Chromosomes were counterstained with DAPI (blue). x-axis, relative DAPI fluorescence; y-axis, relative (GAA)_7_-FITC fluorescence.

**Figure 8:**
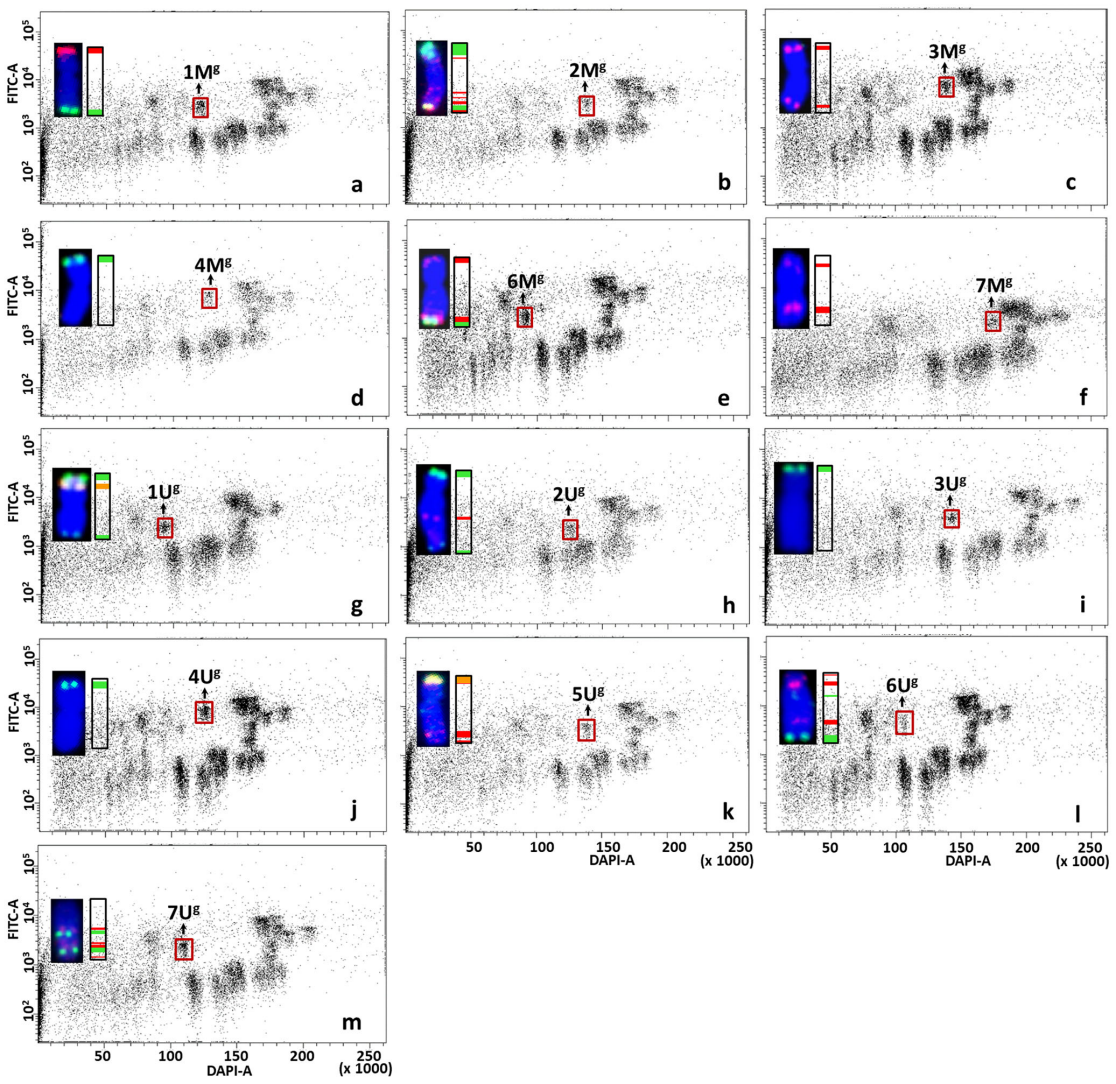
Bivariate flow karyotypes DAPI vs. FITC obtained after FISHIS with GAA_7_ on chromosomes isolated from wheat CS-*Ae. geniculata* acc. TA2899 addition lines. The analysis permitted discrimination of the whole chromosome set from tetraploid *Ae. geniculata* (brown boxes) in the genetic background of bread wheat **(A–M)**. The chromosomes (inset) were sorted in high purity and identified by FISH on microscope slides with probes for *Afa* family repeat (red), 45S rDNA (orange) and *pSc119*.2 repeat (green). Chromosomes were counterstained with DAPI (blue). x-axis, relative DAPI fluorescence; y-axis, relative (GAA)_7_-FITC fluorescence.

Bivariate flow karyotypes of wheat-*Aegilops* addition lines showed that the relative DNA content of *Aegilops* chromosomes was significantly smaller than the B-genome chromosomes but similar to that of A- and D-genome chromosomes. However, as the tetraploid *Aegilops* M- and U-genome chromosomes have prominent (GAA)_7_ clusters, their FITC fluorescence was much higher as compared to wheat A- and D-genome chromosomes. These differences made it possible to discriminate against *Aegilops* chromosomes from those of wheat A, B, and D genomes (**Figures 6**–**8**).

Contrasting bivariate (DAPI-A vs. FITC-A) flow karyotypes of Mv9kr1 (**Figure 5C**) and CS wheats (**Figure 5F**) with their respective wheat-*Aegilops* addition lines (**Figures 6**–**8**), unambiguously confirmed the positions of all alien chromosomes from *Aegilops* on bivariate (DAPI-A vs. FITC-A) dot plots (**Figures 6**–**8**).

*Aegilops* chromosomes were flow sorted from wheat Mv9kr1 and CS addition lines onto microscope slides and could be identified unambiguously by FISH with probes for 45S rDNA, tandem repeats *pSc*119.2 and *Afa* family repeat (**Figures 6**–**8**). FISH analysis revealed that the partial set of M^b^ and U^b^ chromosomes of tetraploid *Ae. biuncialis* could be flow sorted at high purities from wheat Mv9kr1-*Ae. biuncialis* disomic addition lines (2M^b^ 93.4%, 3M^b^ 98.7%, 7M^b^ 79.9%, 1U^b^ 90.2%, 3U^b^ 94.4%, and 6U^b^ 94.1%). Chromosomes 1U^b^ and 6U^b^ were found in a double disomic wheat Mv9kr1-*Ae. biuncialis* addition line (**Figure 7C**) and both chromosomes were sorted separately but simultaneously. The purity data on *Aegilops* chromosomes flow sorted from wheat Mv9kr1-*Ae. biuncialis* addition lines (**Figure 7**) are summarized in **Table 1**.

**Table 1:** Purity in M^b^- and U^b^-genome chromosome fractions flow sorted from Mv9kr1-*Ae. biuncialis* acc. MvGB642 disomic addition lines.

Wheat addition line	Chromosomes	Purity	Contaminating chromosomes	n**
2M^b^ (Mv9kr1-*Ae. biu*)	2M^b^	93.4%	5D: 4.7%, 7D: 5.5%	129
3M^b^ (Mv9kr1-*Ae. biu*)	3M^b^	98.7%	5B: 1.3%	164
7M^b^ (Mv9kr1-*Ae. biu*)	7M^b^	79.9%	5D: 4.2%, 6A: 6.3%, 1A: 4.7%, 2D: 2.1%, 6D: 1.5%, 5A: 1.3%	189
1U^b^ (Mv9kr1-*Ae. biu*)	1U^b^*	90.2%	6U: 9.8%	143
3U^b^ (Mv9kr1-*Ae. biu*)	3U^b^	94.4%	5A: 2.4%, 5D: 1.6%, 5B: 0.8%, 1D: 0.8%	125
6U^b^ (Mv9kr1-*Ae. biu*)	6U^b^*	94.1%	1U: 3.3%, 4D: 1.6%, 6A: 1.0%	120

*Chromosomes found together in one genotype and flow sorted simultaneously but separately.

**Number of classified chromosomes.

The complete set of M^g^- and U^g^-genome chromosomes was successfully flow sorted from wheat CS-*Ae. geniculata* disomic addition lines at high purity (1M^g^–7M^g^: 90.9%, 93.8%, 94.6%, 93.5%, 97.6%, 82.3%, and 96.1%, respectively; 1U^g^–7U^g^: 87.8%, 94.1%, 97.2%, 93.6%, 97.7%, 68.8%, and 90.8%, respectively). The data about the purity of chromosomes flow sorted from wheat CS-*Ae. geniculata* addition lines (**Figure 8**) are summarized in **Table 2**.

**Table 2:** Purity of M^g^ and U^g^ chromosomes flow sorted from CS-*Ae. geniculata* acc. TA2899 disomic addition lines.

Wheat addition line	Chromosome	purity	Contaminating chromosomes	n*
1M^g^ (CS-*Ae. gen*)	1M^g^	90.9%	3A: 4.8%, 2B: 2.0%, 4A: 1.3%, 6B: 1.0%	143
2M^g^ (CS-*Ae. gen*)	2M^g^	93.8%	2D: 2.6%, 4A: 1.7%, 4B: 0.95%, 5B: 0.95%	114
3M^g^ (CS-*Ae. gen*)	3M^g^	94.6%	1B: 3.0%, 4B: 1.5%, 7A: 0.9%	131
4M^g^ (CS-*Ae. gen*)	4M^g^	93.5%	2B: 1.3%, 1B: 1.3%, 6B: 2.5%, 2D: 1.3%	108
5M^g^ (CS-*Ae. gen*)	5M^g^	97.6%	1D: 0.8%, 4A: 1.5%	126
6M^g^ (CS-*Ae. gen*)	6M^g^	82.3%	7BL: 12.9%, 4A: 2.3%, 5D: 1.1%, 4D: 0.7%, 1D: 0.7%	170
7M^g^ (CS-*Ae. gen*)	7M^g^	96.1%	7A: 1.0%, 4A: 1.0%, 2B: 1.9%	103
1U^g^ (CS-*Ae. gen*)	1U^g^	87.8%	7A: 0.9%, 4A: 11.3%	115
2U^g^ (CS-*Ae. gen*)	2U^g^	94.1%	3D: 1.0%, 1D: 3.9%, 5D: 1.0%	102
3U^g^ (CS-*Ae. gen*)	3U^g^	97.2%	1A: 0.56%, 3B: 0.56%, 3D: 0.56%, 1D: 0.56%, 5D: 0.56%	181
4U^g^ (CS-*Ae. gen*)	4U^g^	93.6%	6B: 3.2%, 6A: 1.6%, 5A: 0.8%, 4D: 0.8%	125
5U^g^ (CS-*Ae. gen*)	5U^g^	97.7%	4A: 0.57%, 4D: 0.57%, 6D: 0.57%, 1D: 0.57%	178
6U^g^ (CS-*Ae. gen*)	6U^g^	68.8%	4A: 5.5%, 1D: 17.7%, 6A: 4.4%, 5B: 1.2%, 2D: 1.2%, 3D: 1.2%	110
7U^g^ (CS-*Ae. gen*)	7U^g^	90.8%	4A: 8.4%, 5B: 0.8%	153

*Number of classified chromosomes.

## Discussion

Transfer of genes with agronomical importance from the M and U genomes of *Aegilops* into cultivated wheat could be facilitated by genomic approaches used for pure fractions of individual chromosomes. One approach is the shotgun sequencing of flow sorted chromosomes, which allows one to determine gene content and develop PCR markers for specific cytogenetic positions to support gene introgression processes as demonstrated for diploid *Ae. comosa* and *Ae. umbellulata* (Said et al., 2021). Using bivariate flow cytometric analysis on gene bank accessions and wheat-alien chromosome addition lines, the present study extended the ability of chromosome-based approaches to the allotetraploid *Ae. biuncialis* and *Ae. geniculata.*

Over the years, chromosome flow sorting has proven to be a powerful tool in the analysis of complex plant genomes (Zwyrtková et al., 2021). Among other uses, the method was instrumental in the construction of chromosome-specific DNA libraries and the development of molecular and cytogenetic markers (Šimková et al., 2008a; Doležel et al., 2012; Doležel et al., 2014). The availability of purified chromosome fractions facilitated the establishment of wheat-wild relatives cross-genome homoeology (Molnár et al., 2013; Molnár et al., 2016) and the production of draft sequence assemblies for wild gene source species such as *Ae. comosa*, *Ae. umbellulata* (Said et al., 2021), and *A. cristatum* (Zwyrtková et al., 2022). The method was also essential for the development of reference genome assemblies for cereal species with economic importance, such as barley (International Barley Genome Sequencing Consortium et al., 2012) and bread wheat (International Wheat Genome Sequencing Consortium (IWGSC), 2018), as well as for their wild relative *Ae. sharonensis* (Yu et al., 2022).

### Monovariate flow karyotyping

Flow cytometric analysis of chromosome suspensions from *Ae. biuncialis* and *Ae. geniculata* based on DAPI fluorescence alone showed that some chromosomes could be flow sorted individually (7M^b^ and 1U^b^ from *Ae. biuncialis*; 5M^g^, 6M^g^, and 1U^g^ from *Ae. geniculata* AE1311/00; and 4M^g^, 5M^g^, 6M^g^, and 1U^g^ from *Ae. geniculata* AE660/83). However, the remaining chromosomes could be sorted only into groups of more than two. These observations confirm previous studies carried out on tetraploid *Ae. biuncialis* and *Ae. geniculata*, where monovariate flow karyotypes obtained on DAPI-stained chromosomes showed that two peaks specific to chromosome 7M^b^ and 1U^b^ could be discriminated in tetraploid *Ae. biuncialis* and the chromosomes could be sorted with purities exceeding 95.9% and 81.2%, respectively (Molnár et al., 2011b). The remaining chromosomes formed composite peaks and could only be sorted into groups of six. However, no specific peaks for individual chromosomes were discriminated in tetraploid *Ae. geniculata*. The chromosomes formed composite peaks and could be sorted into groups of two or more (Molnár et al., 2011b). Similar results were obtained in other cultivated and wild cereal species, such as rye, durum wheat, *Ae. umbellulata*, *Ae. comosa*, *Ae. speltoides*, *Ae. markgrafii*, and diploid and tetraploid *A. cristatum* (Kubaláková et al., 2003; Kubaláková et al., 2005; Molnár et al., 2011a; Molnár et al., 2011b; Molnár et al., 2014; Molnár et al., 2015; Said et al., 2019a). This is a common situation in cereals and due to similarity in size, only 1–3 chromosomes show enough difference to form separate peaks, while the remaining chromosomes form either a single composite peak, or several smaller composite peaks (Lysák et al., 1999; Vrána et al., 2000; Kubaláková et al., 2003; Šimková et al., 2008b). Although this result indicates that it is not possible to dissect the whole genome into single chromosomes by size, it shows that it is possible to dissect the complex genome of allotetraploid *Aegilops* species into groups of some specific chromosomes and use these enriched subgenomic fractions for downstream analyses (Vrána et al., 2015).

Monovariate analysis has some significant advantages, such as higher sort yield (typically 400,000–900,000 chromosomes per working day) than bivariate flow sorting (Doležel et al., 2021) and the lack of fluorochrome-labeled DNA in the sorted chromosome fraction, which is required in some downstream applications such as optical mapping. Both of these characteristics allow the purification of high molecular weight (HMW) chromosomal DNA (50–150 kbp). Some genomic approaches, such as BAC library construction (Šafář et al., 2004), optical mapping (Staňková et al., 2016), and chromosome conformation capture (Hi-C) (Mascher et al., 2017), require 600–2,000 ng of HMW DNA, which requires 3–4 weeks of sorting. Monovariate sorting of *Aegilops* chromosomes opens the way for these genomic applications, which facilitate the development of high-quality reference sequences of these chromosomes.

### Bivariate flow karyotyping

Our bivariate flow cytometric analysis indicated that M- and U-genome chromosomes labeled by FISHIS were characterized by high FITC fluorescence intensity, suggesting the presence of large GAA microsatellite clusters in the genomes of *Ae. biuncialis* and *Ae. geniculata*. These observations confirmed the previous results of Molnár et al. (2011a) and Abdolmalaki et al. (2019), who reported the presence of strong GAA signals after FISH on root tip chromosome spreads of *Ae. biuncialis* and *Ae. geniculata.* These results are also in line with the previous findings that the microscopically detectable GAA clusters in the *Aegilops* C, M, N, S, and U genomes can also be visualized by FISH in suspension (FISHIS) to label chromosomes prior to flow cytometric analysis (Giorgi et al., 2013), as it was demonstrated for diploid genome progenitor species (Molnár et al., 2016; Said et al., 2021; Yu et al., 2022). Furthermore, our results on *Ae. biuncialis* and *Ae. geniculata* also suggest that bivariate flow karyotyping on other allopolyploid *Aegilops* species containing M- and U-genomes (i.e., *Ae. columnaris*, *Ae. neglecta*) or the *Aegilops* species with other GAA-rich genome combinations such as S- and U genomes (*Ae. kotschyi*, *Ae. peregrina)* or C- and U genomes (*Ae. triuncialis*) (Kilian et al., 2011) can also be carried out.

Although bivariate flow karyotyping improved the resolution of individual chromosomes relative to monovariate flow karyotyping, it was not sufficient to discriminate all chromosomes of *Ae. biuncialis*, with the exception of 7M^b^ and 1U^b^, which were already flow sorted using univariate flow karyotyping. However, in the case of *Ae. geniculata*, bivariate flow karyotyping allowed separation of nine out of the 14 chromosomes from the accession AE1311/00 with a wild type karyotype (Molnár et al., 2011a). The significant difference in the pattern of bivariate flow karyotype DAPI vs. FITC between *Ae. biuncialis* and *Ae. geniculata* (i.e., differences in chromosome size and GAA abundance) may be related to the structural and functional reorganization processes that lead to genome downsizing after allopolyploidization event (Ozkan et al., 2003; Eilam et al., 2008; Feldman and Levy, 2015). Deletion or proliferation of transposable elements (TE) has been reported as a predominant phenomenon after the allopolyploidization (Leitch et al., 2008). In line with this, some specific TE families, such as *Sabine*, show contrasting evolutionary trajectories after polyploidization, as significant proliferation was observed in *Ae. cylindrica*, while its significant elimination was detected in *Ae. geniculata* relative to diploid genome progenitors (Senerchia et al., 2014). It is feasible that different TE elements were eliminated or proliferated after the formation of *Ae. biuncialis* and *Ae. geniculata*, leading to differences in the size of the individual chromosomes. Sequencing of chromosomes flow sorted from these species and repeat analysis (Tiwari et al., 2015) could clarify the role of TE dynamics in changes of chromosome size following allopolyploidization.

Translocation can modify the size of chromosomes, thereby adding another opportunity to sort individual chromosomes or to prepare subgenomic samples enriched for a particular chromosome. Flow karyotype of *Ae. geniculata* accession AE660/83 having a 2M^g^/5U^g^ reciprocal translocation supports this idea as the translocations T2M^g^S.2M^g^L-5U^g^L and T5U^g^S.5U^g^L-2M^g^L were sorted in purities of 58.2% and 53.8%, respectively, while the non-rearranged chromosomes 2M^g^ and 5U^g^ could be sorted at significantly lower purities (43.7% and 38.2%, respectively) from the *Ae. geniculata* AE1311/00 with normal karyotype. Our results on *Ae. geniculata* are in line with those of Kubaláková et al. (2002), who used the same approach to flow sort translocations T5BL.7BL and T4AL.4AS-5BL from the wheat cultivars Cappelle Desprez and Jubilar, respectively (Kubaláková et al., 2005).

Another approach to overcome the difficulties in flow sorting chromosomes of interest is the use of chromosome addition lines (Doležel and Lucretti, 1995; Doležel et al., 2014). The effectivity of this approach has been demonstrated earlier for the discrimination and flow cytometric sorting of rye chromosomes 2R–7R from wheat–rye disomic addition lines by Kubaláková et al. (2003). Said et al. (2019a) applied this approach to wheat-*A. cristatum* disomic addition lines to flow sort six chromosomes (1P–6P). Interestingly, sorting P-genome chromosomes lacking GAA signals was possible due to the fact that the sizes of *Agropyron* chromosomes were larger than the GAA-poor A-genome chromosomes (2A, 3A, 5A, and 7A) of wheat. The situation was the opposite in the preset work, as the GAA-rich *Aegilops* chromosomes were smaller than the GAA-rich B-genome chromosomes of wheat. Thus, the alien chromosomes were clearly discriminated against those of wheat. We used wheat Mv9kr1-*Ae. biuncialis* acc. MvGB642 and wheat CS-*Ae. geniculata* acc. TA2899 disomic chromosome addition lines, where a partial set of chromosomes of tetraploid *Ae. biuncialis* and a complete set of chromosomes of tetraploid *Ae. geniculata* were individually transferred to bread wheat genotype Mv9kr1 (Schneider et al., 2005) and CS (Friebe et al., 1999), respectively.

DAPI fluorescence intensity (equivalent to chromosome size and relative DNA content) of *Aegilops* chromosomes was lower as compared to wheat B-genome chromosomes and similar to A- and D-genome chromosomes. However, the FITC fluorescence intensity (equivalent to GAA clusters) of M- and U-genome chromosomes was higher as compared to those of wheat A- and D-genomes and lower than B-genome chromosomes, which were strongly labeled by the (GAA)_7_-FITC probe. This permitted discrimination of *Aegilops* chromosomes and allowed sorting of them with purities ranging from 79.9% (7M^b^) to 98.7% (3M^b^) in wheat disomic addition lines Mv9kr1-*Ae. biuncialis* (**Table 1**), and from 68.8% (6U^g^) to 97.7% (5U^g^) in wheat disomic addition lines CS-*Ae. geniculata* (**Table 2**). A slightly lower purity of 6U^g^ could be explained by its small size and overlap of its population on flow karyotype with the region representing the smallest wheat chromosomes 1D, 4D, and 6D (**Table 2**). A similar phenomenon was reported by Said et al. (2019a), where the low purities of *A. cristatum* telocentric chromosomes flow sorted from wheat-*A. cristatum* ditelosomic addition lines were attributed to the fact that the flow karyotype position of P-genome telosomes overlapped with the region of wheat chromosome fragments.

In this work, applying bivariate analysis enabled the dissection of M- and U-genomes into individual chromosomes, each of them representing only 12.8% (1M)–15.2% (3M) and 12.7% (1U)–16.0% (4U) of the M- and U-genomes, respectively (Said et al., 2021). The ability to purify individual chromosomes from tetraploid *Ae. biuncialis* and *Ae. geniculata* opens the way for some important genomic applications. The DNA of flow sorted chromosomes is suitable for Illumina sequencing to get insights into their repeat landscape and gene content (Tiwari et al., 2015; Zwyrtková et al., 2021) and thereby makes it possible to carry out comparative phylogenomic analysis of diploid and tetraploid species of *Aegilops* as well as wheat-*Aegilops* genome comparisons.

It is known that *ph*-induced meiotic chromosome pairing is one of the most important methods for homoeologous gene transfer from wild relatives into wheat (Qi et al., 2007). Meiotic chromosome pairing analysis of *T. aestivum × Ae. geniculata* F_1_ hybrids indicated that M- and U-genome chromosomes of *Aegilops* are pairing predominantly with A- and D- genome chromosomes of hexaploid wheat, while their pairing with B-genome chromosomes is extremely rare (Cifuentes et al., 2006; Cifuentes and Benavente, 2009). This chromosome pairing behavior suggests that GAA-rich M- and U-genome chromosome segments can be introgressed most probably into the GAA-poor A- or D- genomes of wheat (Bansal et al., 2020; Steadham et al., 2021). A-*Aegilops* or D-*Aegilops* translocations developed in the future will presumably be suitable for flow sorting because of their stronger GAA-FITC signal as compared to normal A- or D-genome chromosomes. If so, a chromosome-based approach can be effectively used to identify introgressed genes with agronomic importance (Zwyrtková et al., 2021). Recently developed gene cloning methods such as “Targeted chromosome-based cloning *via* long-range assembly” (TACCA) (Thind et al., 2017) and “Mutagenesis Chromosome flow sorting and short-read sequencing” (MutChromSeq) (Sánchez-Martín et al., 2016) provide opportunities to identify genes transferred from wild relatives into wheat (Xing et al., 2018).

Finally, we can conclude that the applied bivariate flow cytometric analysis made it possible to dissect the complex genomes of allotetraploid *Ae. biuncialis* and *Ae. geniculata* into individual chromosomes. The chromosome specific subgenomic DNA samples provide attractive resources to perform structural and functional genome analysis of these important wild gene sources of wheat and to develop molecular tools to facilitate the exploitation of tetraploid *Ae. biuncialis* and *Ae. geniculata* in wheat-alien introgression breeding.

## Data availability statement

The original contributions presented in the study are included in the article/**Supplementary Material**. Further inquiries can be directed to the corresponding author.

## Author contributions

The work involved collaboration between all authors. BF, JD, IM, MS, and PC conceived research theme and discussed results. MS and PC performed lab work and flow sorted the chromosomes. IM and MS designed the experiments, carried out FISH tests and drafted the manuscript. AF, EG, IM, and LI carried out the purity estimations and calculations. All authors contributed to the article and approved the submitted version.

## Funding

This work has been supported by the ERDF project ‘Plants as a tool for sustainable global development’ (No. CZ.02.1.01/0.0/0.0/16_019/0000827), the Hungarian National Research, Development and Innovation Office (K135057, TKP2021-NKTA-06, and 2019-2.1.11-TÉT-2019-00074), the Marie Curie Fellowship Grant award ‘AEGILWHEAT’ (H2020-MSCA-IF-2016-746253), and the ELIXIR-CZ project (LM2015047), a component of the international ELIXIR infrastructure that is part of the project “e-Infrastruktura CZ” (LM2018140) within the program Projects of Large Research, Development and Innovations Infrastructures.

## Acknowledgments

The authors thank Dr. John Raupp from the Kansas State University, Manhattan, Kansas, USA for helping with providing the seeds of wheat cv. CS and CS-*Ae. geniculata* accession TA2899 chromosome addition lines 1M^g^–7M^g^ and 1U^g^–7U^g^. We appreciate technical assistance of Zdeňka Dubská, Romana Šperková, and Jitka Weiserová.

## Conflict of interest

The authors declare that the research was conducted in the absence of any commercial or financial relationships that could be construed as a potential conflict of interest.

## Publisher’s note

All claims expressed in this article are solely those of the authors and do not necessarily represent those of their affiliated organizations, or those of the publisher, the editors and the reviewers. Any product that may be evaluated in this article, or claim that may be made by its manufacturer, is not guaranteed or endorsed by the publisher.
