# Supplementary material for: Flow karyotyping of wheat-Aegilops additions facilitate dissecting the genomes of Ae. biuncialis and Ae. geniculata into individual chromosomes
Source: Front Plant Sci. 2022 Oct 3;13:1017958. doi: 10.3389/fpls.2022.1017958 (PMC9575658; doi:10.3389/fpls.2022.1017958)
Supplement: Supplementary file 1 [file DataSheet_1.docx]

Supplementary Material

# Supplementary Figures


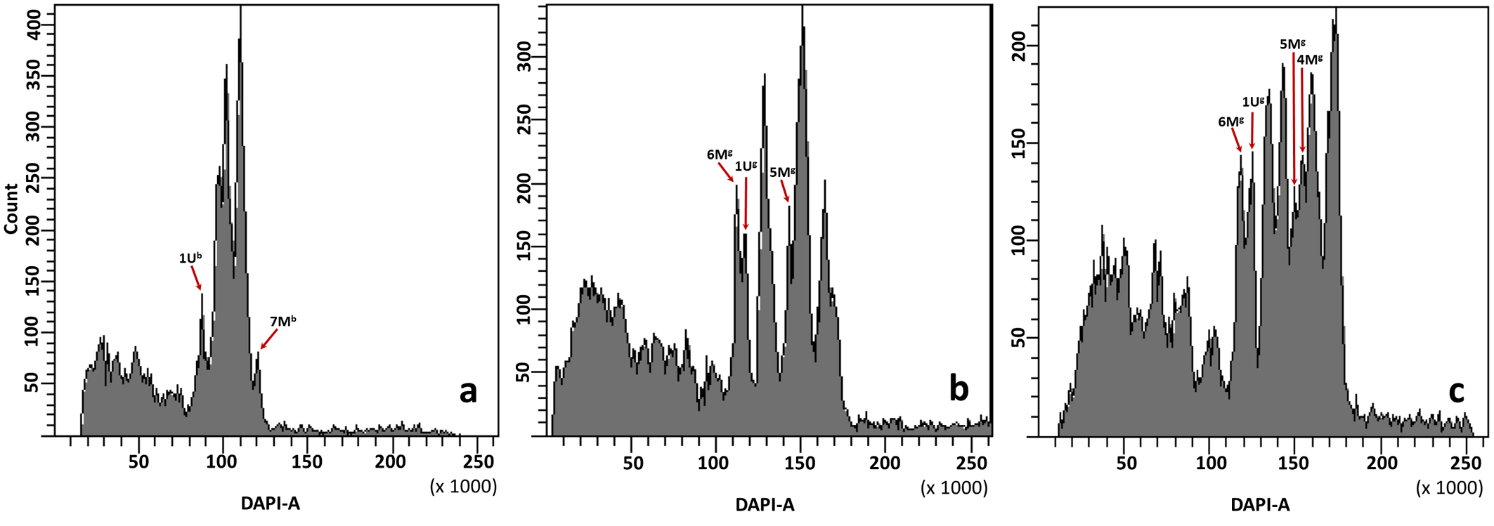


**Supplementary Figure 1.** Monovariate flow cytometric analysis of tetraploid *Aegilops*. Flow karyotypes of *Ae. biuncialis* MvGB382 (a), *Ae. geniculata* 1311/00 (b) and *Ae. geniculata* AE660/83 (c) showing the possibility of sorting individual chromosomes (brown arrows) using chromosome size/DNA content (x-axis) based on DAPI fluorescence, y-axis shows count.
