# Supplementary material for: Flow karyotyping of wheat-Aegilops additions facilitate dissecting the genomes of Ae. biuncialis and Ae. geniculata into individual chromosomes
Source: Front Plant Sci. 2022 Oct 3;13:1017958. doi: 10.3389/fpls.2022.1017958 (PMC9575658; doi:10.3389/fpls.2022.1017958)
Supplement: Supplementary file 2 [file Table_1.docx]

Supplementary Material

# Supplementary Tables

**Supplementary Table 1.** Accession number, genome formula and the source genebank of the plant material used in the present study.

| **Genotype** | **Accession** | **Genome** | **Source^1^** |
| --- | --- | --- | --- |
| *Ae. biuncialis* | MvGB382 | U^b^U^b^M^b^M^b^ | MvGB |
| *Ae. geniculata* | AE1311/00 | U^g^U^g^M^g^M^g^ | IPK |
| *Ae. geniculata* | AE660/83 | U^g^U^g^M^g^M^g^ | IPK |
| *T. aestivum* | Mv9kr1 | AABBDD | MvGB |
| *T. aestivum* | Chinese Spring | AABBDD | MvGB |
| *T.aestivum*/*Ae. geniculata* 1U^g^ addition | TA 7662 | AABBDD+1U^g^1U^g^ | WGRC |
| *T.aestivum*/*Ae. geniculata* 2U^g^ addition | TA 7663 | AABBDD+2U^g^2U^g^ | WGRC |
| *T.aestivum*/*Ae. geniculata* 3U^g^ addition | TA 7664 | AABBDD+3U^g^3U^g^ | WGRC |
| *T.aestivum*/*Ae. geniculata* 4U^g^ addition | TA 7665 | AABBDD+4U^g^4U^g^ | WGRC |
| *T.aestivum*/*Ae. geniculata* 5U^g^ addition | TA 7666 | AABBDD+5U^g^5U^g^ | WGRC |
| *T.aestivum*/*Ae. geniculata* 6U^g^ addition | TA 7667 | AABBDD+6U^g^6U^g^ | WGRC |
| *T.aestivum*/*Ae. geniculata* 7U^g^ addition | TA 7688 | AABBDD+7U^g^7U^g^ | WGRC |
| *T.aestivum*/*Ae. geniculata* 1M^g^ addition | TA 7655 | AABBDD+1M^g^1M^g^ | WGRC |
| *T.aestivum*/*Ae. geniculata* 2M^g^ addition | TA 7656 | AABBDD+2M^g^2M^g^ | WGRC |
| *T.aestivum*/*Ae. geniculata* 3M^g^ addition | TA 7657 | AABBDD+3M^g^3M^g^ | WGRC |
| *T.aestivum*/*Ae. geniculata* 4M^g^ addition | TA 7658 | AABBDD+4M^g^4M^g^ | WGRC |
| *T.aestivum*/*Ae. geniculata* 5M^g^ addition | TA 7659 | AABBDD+5M^g^5M^g^ | WGRC |
| *T.aestivum*/*Ae. geniculata* 6M^g^ addition | TA 7660 | AABBDD+6M^g^6M^g^ | WGRC |
| *T.aestivum*/*Ae. geniculata* 7M^g^ addition | TA 7661 | AABBDD+7M^g^7M^g^ | WGRC |
| *T.aestivum*/*Ae. biuncialis* 1U^b^/6U^b^ double addition | - | AABBDD+1U^b^1U^b^6U^b^6U^b^ | MvGB |
| *T.aestivum*/*Ae. biuncialis* 3U^b^ addition | - | AABBDD+3U^b^3U^b^ | MvGB |
| *T.aestivum*/*Ae. biuncialis* 2M^b^ addition | - | AABBDD+2M^b^2M^b^ | MvGB |
| *T.aestivum*/*Ae. biuncialis* 3M^b^ addition | - | AABBDD+3M^b^3M^b^ | MvGB |
| *T.aestivum*/*Ae. biuncialis* 7M^b^ addition | - | AABBDD+7M^b^7M^b^ | MvGB |

^1^MvGB, accessions from the Martonvásár Cereal Genebank (Martonvásár, http://mgi.atk.hu/); TA, accessions from the Wheat Genetics Resource Center, WGRC (Kansas, KS, United States, <https://www.k-state.edu/wgrc/genetic_resources>) and AE, accessions from the Leibniz Institute of Plant Genetics and Crop Plant Research, IPK (Gatersleben, Germany, <https://www.ipk-gatersleben.de/en/infrastructure/gene-bank>)
